# Pancreatic neuroendocrine carcinoma with unique morphological features mimicking intraductal papillary mucinous carcinoma: A case report

**DOI:** 10.3389/fmed.2022.951834

**Published:** 2022-07-13

**Authors:** Hidekazu Tanaka, Kosuke Minaga, Yasuo Otsuka, Yasuhiro Masuta, Ken Kamata, Kentaro Yamao, Mamoru Takenaka, Tomoko Hyodo, Masatomo Kimura, Tomohiro Watanabe, Masatoshi Kudo

**Affiliations:** ^1^Department of Gastroenterology and Hepatology, Kindai University Faculty of Medicine, Osaka-Sayama, Japan; ^2^Department of Radiology, Kindai University Faculty of Medicine, Osaka-Sayama, Japan; ^3^Department of Pathology, Kindai University Faculty of Medicine, Osaka-Sayama, Japan

**Keywords:** pancreatic neuroendocrine carcinoma, neuroendocrine neoplasm, neuroendocrine carcinoma, per-oral pancreatoscopy, pancreatoscopy

## Abstract

**Background:**

Pancreatic neuroendocrine carcinoma (PanNEC) is a rare disease entity with rapid progression and poor prognosis. Here, we report a PanNEC case with unique morphological features mimicking intraductal papillary mucinous carcinoma.

**Case presentation:**

A 69-year-old Japanese man was referred to our hospital for further evaluation of weight loss and deterioration of diabetes mellitus. Contrast-enhanced computed tomography showed a solid and cystic mass with hypo-enhancement at the tail of the pancreas. The main pancreatic duct (MPD) was diffusely dilated without obstruction, accompanied by marked parenchymal atrophy. Multiple peritoneal and omental nodules were observed, suggesting tumor dissemination. Endoscopic retrograde cholangiopancreatography revealed that the mass correlated with the dilated MPD. During pancreatography, a large amount of mucus was extruded from the pancreatic orifice of the ampulla. Based on these imaging findings, intraductal papillary mucinous carcinoma was suspected. Per-oral pancreatoscopy (POPS)-guided tumor biopsies were conducted for the lesion's solid components. Histopathological examination of the biopsied material confirmed small-cell-type PanNEC with a Ki-67 labeling index of 90%. Due to his condition's rapid decline, the patient was given the best supportive care and died 28 days after diagnosis.

**Conclusion:**

Although rare, PanNEC, which correlates with the MPD and is accompanied by marked dilation of the MPD, does exist as one phenotype. In such cases, POPS-guided biopsy could be a useful diagnostic modality.

## Introduction

Pancreatic neuroendocrine neoplasm (PanNEN) is a rare type of pancreatic neoplasm, accounting for 1%−2% of pancreatic tumors (1). According to the World Health Organization (WHO) classification of 2019, PanNENs are classified as follows based on the degree of tumor cell differentiation and proliferation activity assessed by the Ki-67 labeling index: well-differentiated pancreatic neuroendocrine tumor (PanNET), poorly differentiated pancreatic neuroendocrine carcinoma (PanNEC), and mixed neuroendocrine-non-neuroendocrine neoplasm (2, 3). Treatment strategy and prognosis closely depend on the WHO classification. PanNEC is characterized by a poorly differentiated and high Ki-67 labeling index, accounting for only 2%−3% of all PanNENs with a uniformly poor prognosis (4, 5). Although little has been elucidated about PanNENs molecular drivers, a recent comprehensive genomic analysis revealed that PanNECs are genetically distinct from PanNETs (6).

Due to its rarity and non-specific imaging findings, PanNEC is sometimes difficult to consider as a differential diagnosis for various pancreatic neoplasms; therefore, histopathological examination is mandatory for confirmatory diagnosis. Endoscopic ultrasonography-guided tissue acquisition (EUS-TA) has been adopted as a first-line procedure for the definitive histological diagnosis of pancreatic lesions (7, 8). Recently, per-oral pancreatoscopy (POPS)-guided tissue biopsy has evolved as another diagnostic modality for pancreatic lesions located in or communicating with the main pancreatic duct (MPD) (9, 10).

Here, we report a PanNEC case with cystic degeneration masquerading as intraductal papillary mucinous carcinoma (IPMC), which was successfully diagnosed by direct tumor biopsy under POPS.

## Case presentation

**Figure 1:**
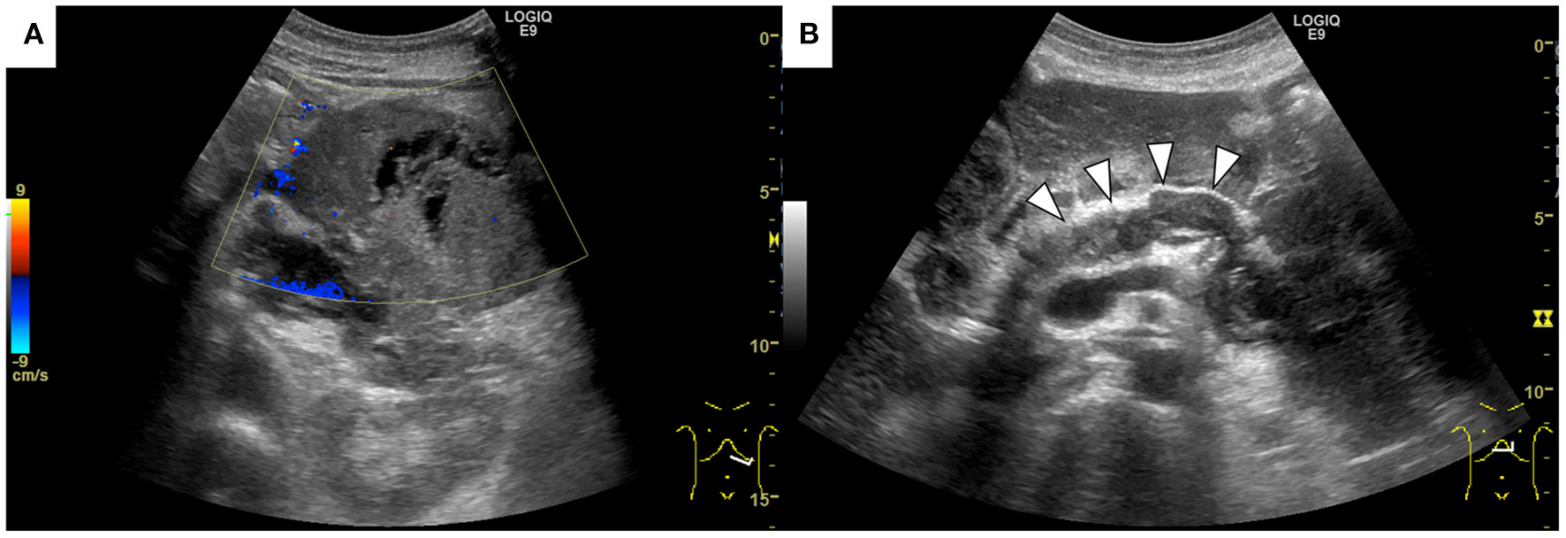
Abdominal ultrasonography shows a well-defined, irregular-shaped, heterogeneous mass at the pancreatic tail **(A)**. The main pancreatic duct was dilated with hyperechoic structures inside (arrowheads) **(B)**.

**Figure 2:**
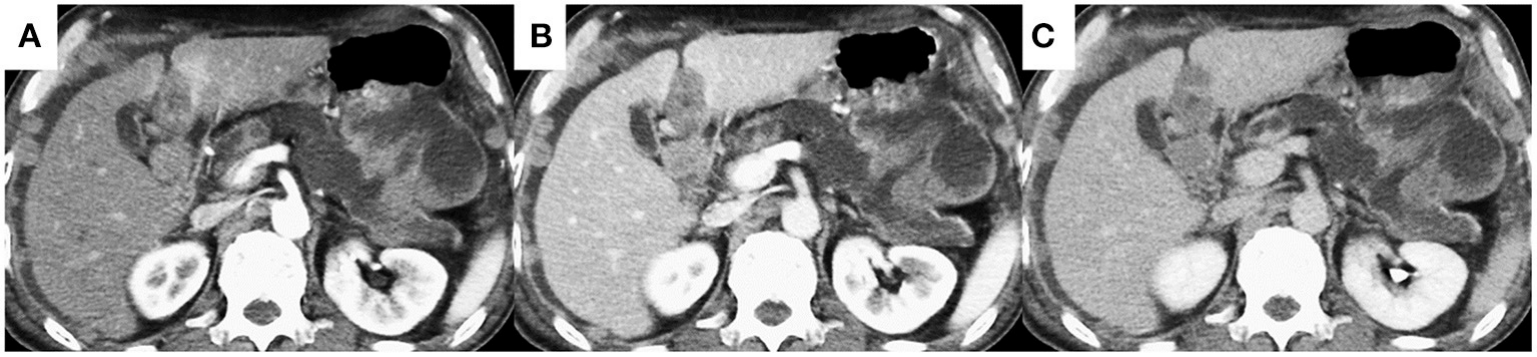
Dynamic contrast-enhanced computed tomography shows that the pancreatic tail mass has solid and cystic components. The solid components of the lesion showed hypo-enhancement, whereas the cystic components of the lesion showed non-enhancement during all phases **(A)** arterial phase, **(B)** portal phase, **(C)** equilibrium phase).

**Figure 3:**
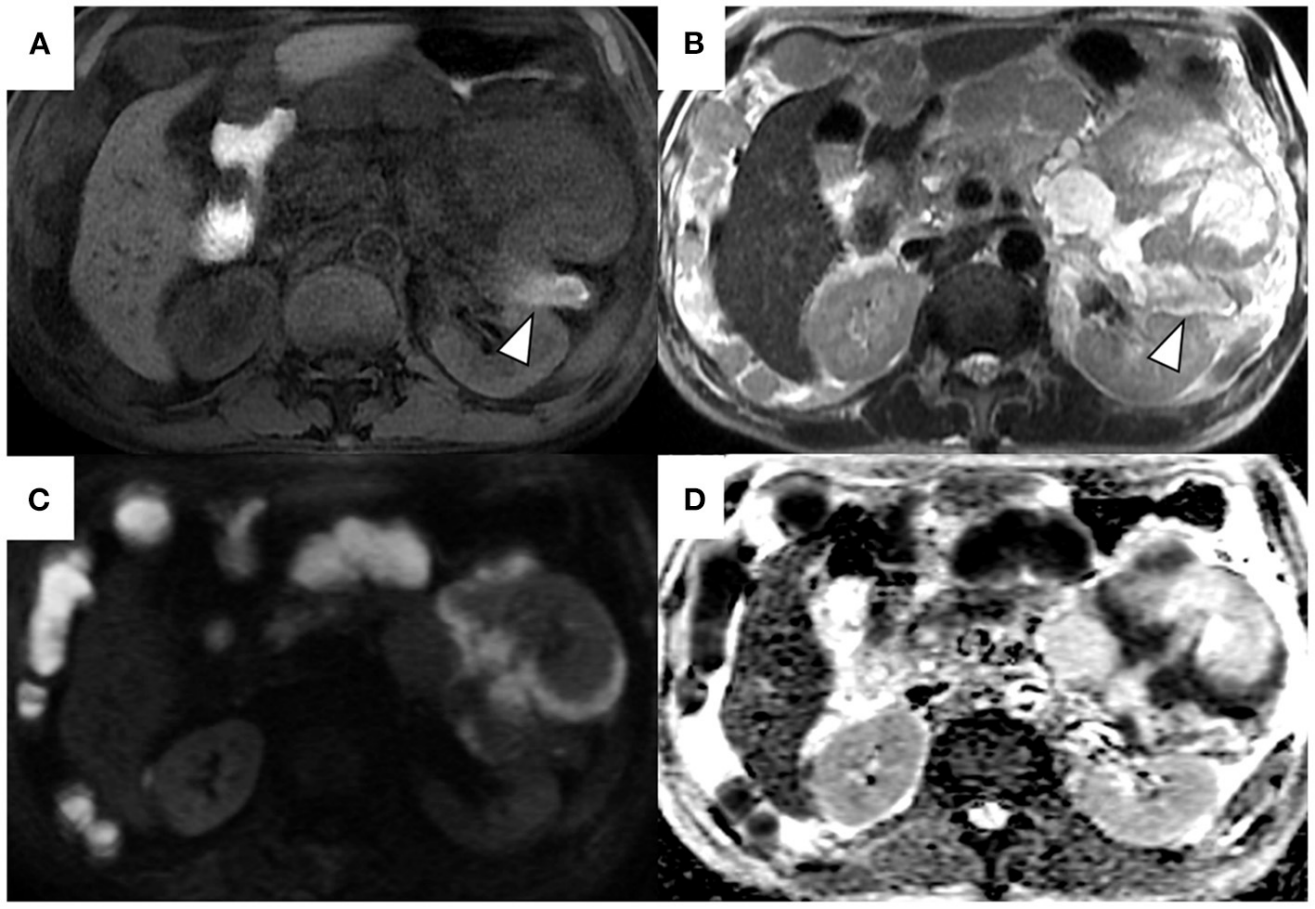
Magnetic resonance T1-weighted image **(A)**, T2-weighted image **(B)**, diffusion-weighted image **(C)**, and apparent diffusion coefficient map **(D)**. T2-weighted images show a heterogeneous and hyperintense mass in the pancreatic tail **(B)**. The area corresponding to solid components on computed tomography shows hyperintensity on diffusion-weighted images with a b factor of 800 s/mm2 and hypointensity on the apparent diffusion coefficient map **(C,D)**. The signal intensity of the main pancreatic duct in the tail was high on T1-weighted image and low on the T2-weighted image (arrowheads).

**Figure 4:**
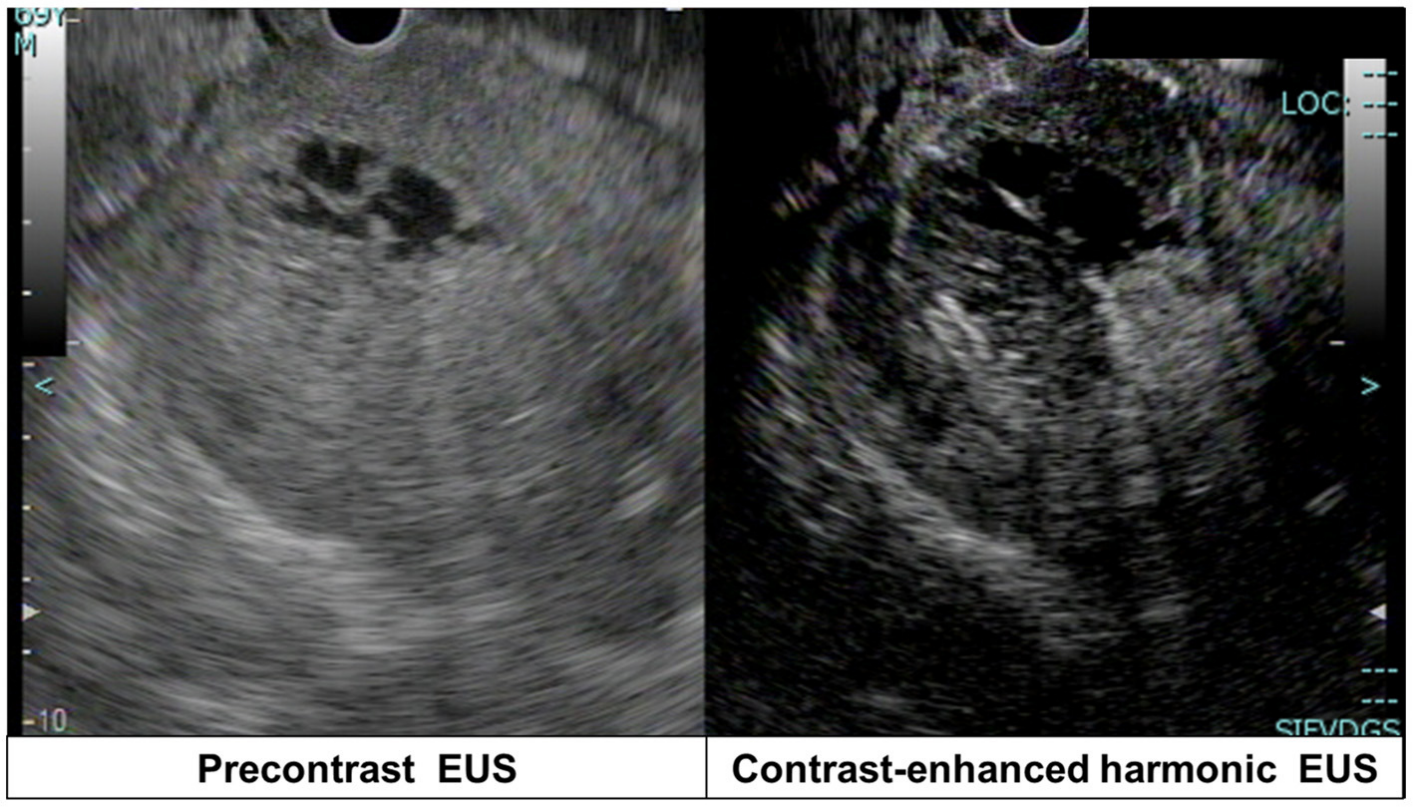
Contrast-enhanced harmonic endoscopic ultrasonography shows mixed hypo-/non-enhancement patterns in the pancreatic tail mass solid components.

A 69-year-old Japanese man was admitted to our hospital for further evaluation of weight loss and deterioration of diabetes mellitus. He was a smoker as well a social drinker with no signs of obesity; he had no history of pancreatic disorders. His latest serum HbA1c level had risen to 9.8%. His vital signs were as follows: body temperature of 37.6°C, blood pressure of 146/80 mmHg, pulse rate of 80 beats/min, respiratory rate of 18/min, and oxygen saturation of 98% on room air temperature. Physical examination revealed mild left-upper quadrant tenderness without rebound tenderness. Laboratory tests showed that a complete blood count and coagulation function were unremarkable. Serum biochemistry analysis revealed increased lactic dehydrogenase levels (LDH; 1118 U/L), C-reactive protein (CRP; 5.16 mg/dL), and decreased total protein and albumin levels. Tumor markers showed a mild elevation in carcinoembryonic antigen (CEA; 7.5 ng/mL), carbohydrate 19–9 (CA19–9; 109 U/mL), and a marked elevation in neuron-specific enolase (NSE; 270 ng/mL; normal value: <16.2 ng/mL). The level of duke pancreatic monoclonal antigen type 2 (DUPAN-2) was within the normal limits. Abdominal ultrasonography showed a well-defined, irregular-shaped, heterogeneous mass at the pancreatic tail (Figure 1A). The MPD was dilated with hyperechoic structures (Figure 1B). Abdominal precontrast computed tomography (CT) showed a 97 × 77-mm irregular hypodense mass in the pancreatic tail. Ascites was noted around the liver and pelvis. Dynamic contrast-enhanced CT revealed that the mass in the pancreatic tail had solid and cystic components. The solid components of the lesion showed hypo-enhancement, whereas the cystic components of the lesion showed non-enhancement during all phases (Figures 2A–C). The MPD was diffusely dilated up to 27 mm (predominantly in the tail) without both obstruction and wall thickness, accompanied by marked atrophy of the pancreatic parenchyma. Multiple, well-defined, predominantly solid nodules were observed in the peritoneum and omentum, suggesting tumor dissemination. Following magnetic resonance imaging (MRI) (Figures 3A–D), T2-weighted images showed a heterogeneous and hyperintense mass in the pancreatic tail (Figure 3B). The area corresponding to the solid component seen on CT showed hyperintensity on diffusion-weighted images with b factor of 800 s/mm^2^ and hypointensity on the apparent diffusion coefficient map, indicating diffusion restriction (Figures 3C,D). The signal intensity of the MPD in the tail was high on the T1-weighted image and low on the T2-weighted image (Figures 3A,B), suggesting the presence of a highly viscous substance like mucin within the MPD. The correlation between the cystic components of the lesion and MPD was suggested but not confirmed following CT and MRI. The MRI signal pattern of peritoneal and omental nodules was similar to the solid component of the pancreatic mass. Contrast-enhanced harmonic EUS (CH-EUS), the solid component of the pancreatic tail tumor, showed mixed hypo- and non-enhancement patterns (Figure 4). Additionally, the hyperechoic lesions in the MPD showed non-enhancement, suggesting mucus clots. EUS-TA for the pancreatic mass was not performed owing to the intervening cystic components of the tumor and the avascular areas suggesting tumor necrosis.

**Figure 5:**
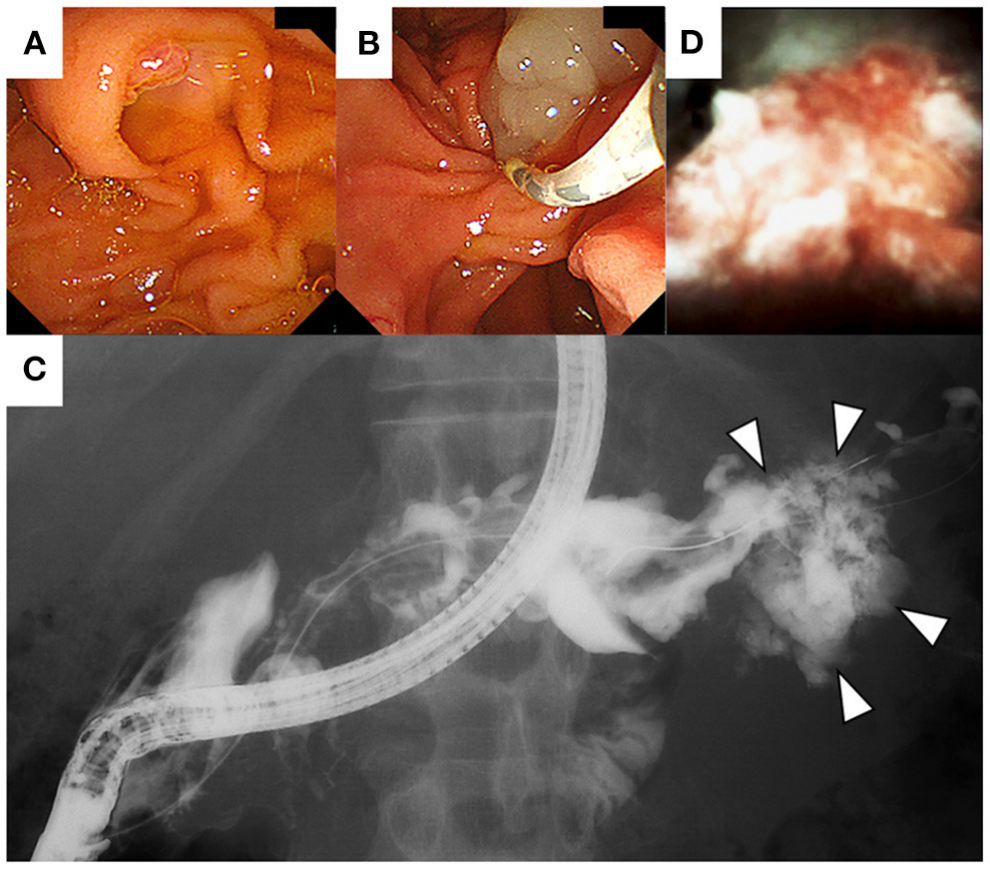
On endoscopic retrograde cholangiopancreatography (ERCP), the papillae shows a fish-mouth appearance **(A)**. After ERCP catheter insertion into the main pancreatic duct, extrusion of mucus from the pancreatic orifice of the papillae was observed **(B)**. Contrast injection shows the correlation between the main pancreatic duct and the mass at the pancreatic tail **(C)**. Per-oral pancreatoscopy showing an irregular-shaped solid mass in the pancreatic tail **(D)**.

**Figure 6:**
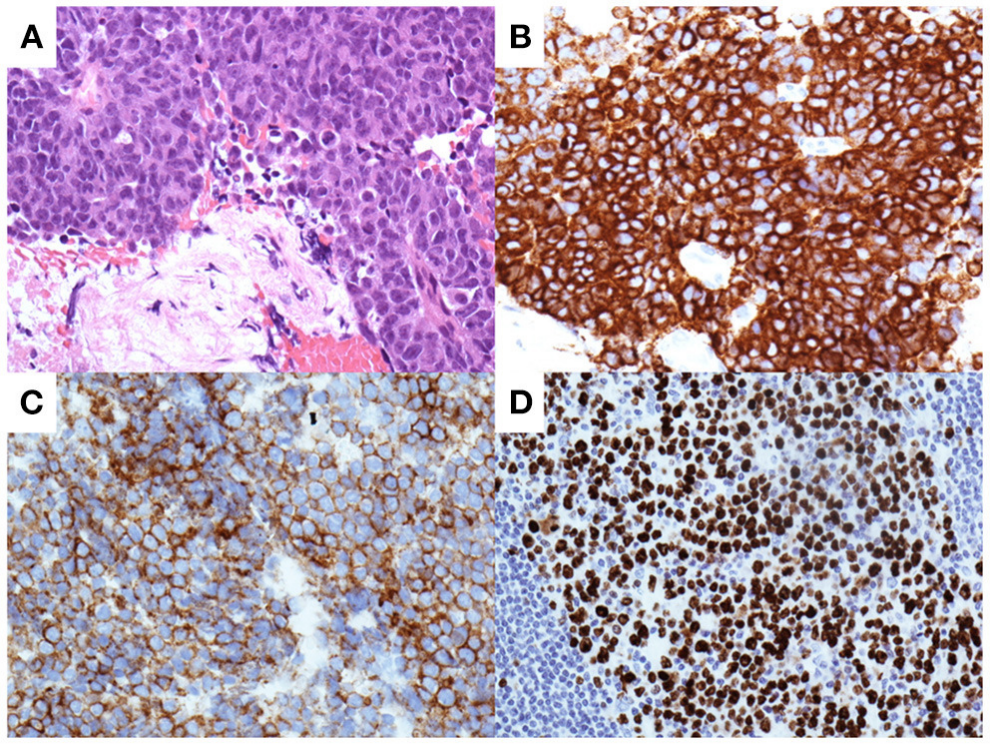
Pathological analysis of the pancreatic tissues obtained by biopsy with per-oral pancreatoscopy. Hematoxylin and eosin staining show a marked proliferation of small round atypical cells with hyperchromatic nuclei and scanty cytoplasm **(A)**. Immunohistochemical analysis shows tumor cells positive for synaptophysin **(B)** and CD56 **(C)**. Tumor cells demonstrate a Ki-67 labeling index of 90% **(D)**.

The features of the pancreatic tumor with cystic components that were communicated with the MPD favored the possible diagnosis of IPMC. Since the MPD was diffusely dilated, POPS-guided direct tumor biopsy under endoscopic retrograde cholangiopancreatography (ERCP) guidance was planned to confirm the histological diagnosis. The ampullary orifice was dilated to the so-called fish-mouth appearance (Figure 5A). After ERCP catheter insertion into the MPD, extrusion of highly viscous mucus from the pancreatic orifice of the ampulla was observed (Figure 5B). Contrast injection during ERCP confirmed the correlation between the MPD and the mass at the pancreatic tail (Figure 5C). Pancreatography revealed that the MPD was filled with contrast defects, suggesting large amounts of mucus. A pancreatoscope (SpyGlass DS, Boston Scientific Corporation, Marlborough, MA, USA) was inserted into the pancreatic tail along with the guidewire kept in the MPD. The wall of the MPD was smooth and no papillary lesions suggestive of mucinous neoplasm were observed. POPS revealed an irregular-shaped solid mass in the pancreatic tail after the removal of the abundant and highly viscous mucin (Figure 5D). Visually directed biopsies were performed from the reddish and raised part of the mass in the pancreatic tail to confirm the histological diagnosis. Histopathological examination of the biopsied material with hematoxylin and eosin staining showed a marked proliferation of small round atypical cells with hyperchromatic nuclei and scanty cytoplasm (Figure 6A). Immunohistochemical analysis showed that the constituent cells were positive for synaptophysin (Figure 6B), chromogranin A, and CD56 (Figure 6C) and negative for leukocyte common antigen, MUC1, MUC2, and MUC5AC. They had a Ki-67 labeling index of 90% (Figure 6D). The findings on pathology and immunohistochemistry suggested the diagnosis of small-cell-type PanNEC.

Due to the patient's rapid deterioration, chemotherapy could not be initiated. Although the best supportive care was given to the patient, he died 28 days after the diagnosis.

## Discussion and conclusion

We reported a case of PanNEC with interesting imaging findings mimicking IPMC. Based on their morphological characteristics, pancreatic tumors are roughly classified into solid and cystic neoplasms. PanNECs are typically solid neoplasms, whereas IPMCs are some of the most common pancreatic cystic tumors; thus, imaging findings of the two are quite different. However, PanNECs can cause cystic change, which sometimes makes it difficult to distinguish from other pancreatic cystic tumors, as in this case. The frequency of cystic change in PanNEN is about 11%−17% (11–13). It has been theorized that cyst development in PanNEN is related to cystic degeneration secondary to tumor necrosis or intralesional hemorrhage due to vascular disruption (13, 14). For PanNEN with cystic degeneration due to tumor necrosis, cyst development is usually observed within the solid neoplasm. No reports of PanNEN with fluid retention causing marked dilation of the MPD have been described; therefore, it seems difficult to establish that the significant fluid retention in this case is only caused by tumor necrosis. Although, some previous studies have suggested that PanNENs with cystic degeneration are associated with a more favorable prognosis (12, 13), PanNEC, which progresses rapidly and has a poor prognosis, as in this case, is also included in cystic PanNENs. The clinical significance of cystic degeneration of PanNEC needs to be investigated in a larger number of cases.

One question that arises from this case is whether the large amount of highly viscous fluid in the MPD was produced from the PanNEC tumor cells. Conceptually, since PanNENs do not produce mucin, two major hypotheses are considered: 1) coexistence of other mucus-producing tumors represented by intraductal papillary mucinous neoplasm (IPMN) or 2) trans-differentiation to neuroendocrine cells from other mucin-producing tumor cells. As for the former hypothesis, although whether the coexistence of IPMN and PanNEN is a real association or a coincidence remains unclear, the association between the two neoplasms has been increasingly reported (15, 16). To obtain histological evidence supporting these hypotheses, we analyzed the mucin expression characteristic of IPMN in pancreatic tissues collected by POPS-guided biopsy; however, none of these mucin expressions were positive. Additionally, abdominal CT obtained 2 years before admission showed no pancreatic cystic tumors and MPD dilation, suggesting that the coexistence of IPMN and PanNEC, in this case, was negative. Regarding the latter hypothesis, recently, an interesting case of mixed IPMN and PanNEN with the same KRAS, GNAS, and CDKN2A mutations and cyclin D1 gene amplification has been reported, supporting the existence of a common progenitor cell of both neoplasms (17). However, because biopsy samples cannot cover the entire tumor, it could not be fully evaluated whether the tumor was composed of PanNEC components only or mixed PanNEC and IPMN components. In this case, the obtained tissue did not contain any IPMN components; therefore, histological evaluation of the entire tumor with surgical specimens would have been necessary to prove the latter hypothesis. Additionally, molecular investigations of the tumor cells may provide hints to prove the latter hypothesis because major molecular alterations differ between these neoplasms (17).

Regarding radiological findings, PanNECs are characterized by hypo-enhancing masses that show heterogeneous enhancement, sometimes accompanied by cystic changes, calcification, and necrosis (18). Following MRI, PanNECs typically show low signal intensity on T1-weighted images and moderately high signal intensity on T2-weighted images compared with the pancreatic parenchyma and are associated with lower apparent diffusion coefficient values in diffusion-weighted images (18). In this case, radiological images of solid components of the pancreatic mass were consistent with the characteristics of the abovementioned imaging findings; however, these findings are not specific to PanNEC and are commonly observed in other pancreatic malignancies such as pancreatic ductal adenocarcinoma. As a more specific imaging finding in this case, the correlation between the tumor and the MPD and diffuse dilation of the MPD was observed; these findings support IPMC rather than PanNEC. The clinical diagnosis of IPMC was also supported by the fish-mouth appearance of the main papillae and the highly viscous fluid retention in the MPD detected during ERCP.

Currently, EUS is considered a well-established diagnostic modality for differentiating pancreatic tumors because of its high-resolution images obtained in real time (19). Notably, the development of new image enhancement technologies, such as CH-EUS and EUS elastography, has improved the characterization of pancreatic tumors (20, 21). A recently published meta-analysis showed that the pooled estimates of sensitivity and specificity for diagnosing pancreatic cancer were 93% and 80%, respectively (22). Although these image enhancement technologies are useful for differentiating between benign and malignant pancreatic tumors, their usefulness in PanNEC is unelucidated because they have not been investigated in many cases. Although obtaining pancreatic tissues is another advantage of EUS, EUS-TA was not performed in this case. Since the possibility of needle tract seeding in a case of IPMC was first reported by Hirooka et al. (23), EUS-FNA has not been actively performed for cystic pancreatic lesions especially in Japan (24). Additionally, EUS-TA has some limitations for making a definite diagnosis when sufficient tissue volume is required for immunostaining or Ki-67 labeling index, as in the PanNEN cases. Recently, we reported that the presence of avascular areas within pancreatic tumors on CH-EUS is negatively associated with the diagnostic sensitivity of EUS-TA (25). Because avascularity was observed in about one-third of the tumor on CH-EUS in this case, it would have been challenging to reach an accurate diagnosis of PanNEC with samples obtained by EUS-TA.

Visually directed biopsy with POPS is another modality for the histological diagnosis of pancreatic tumors originating from the MPD and tumors communicating with the MPD. A recent study that included 78 cases reported that the sensitivity, specificity, and diagnostic accuracy values for visually directed biopsies under POPS guidance were 91%, 95%, and 94%, respectively (26). Although POPS has been increasingly used to evaluate suspected pancreatic duct neoplasia with the advent of SpyGlass DS, a catheter-based single-operator pancreatoscopy, POPS insertion remains technically difficult in patients with non-dilated MPD or severe angulation. Additionally, the risk of post-POPS-pancreatitis is reported to be higher in such patients (27, 28). Although the application of POPS is limited to high-volume expert centers due to the procedural complexity and high cost, pancreatic tumors with MPD dilation seem to be a good indication of POPS for providing direct macroscopic assessment and targeted tissue acquisition. Considering the difference in tissue sampling techniques, compared to EUS-TA, POPS-guided tissue biopsy may provide high-quality specimens with preserved tissue structure and less blood contamination; however, confirmation of this idea awaits future studies that directly compare the diagnostic abilities of both modality for pancreatic lesions.

For managing PanNECs, surgery is the first-line treatment for resectable cases; however, PanNECs are frequently diagnosed at the advanced stage with distant metastases. A Japanese nationwide survey showed that distant metastasis during diagnosis was observed in 46% of PanNEC cases (29). Although the prognosis of PanNECs is poor regardless of the treatment, a recent analysis using the American National Cancer Database has found that surgical resection was strongly and independently associated with improved overall survival of PanNECs (30). In that study, PanNECs had a survival advantage when treated with surgery (median overall survival of 29 months with surgery *vs*. 7 months without surgery) (30). In recent years, the efficacy of EUS-guided radiofrequency ablation or ethanol injection therapy has been reported as an alternative to surgery for local treatment of PanNENs (31, 32). Regarding distant metastasis, the liver is the most common metastatic organ, and hepatectomy for single liver metastasis or transcatheter arterial embolization for multiple liver metastases may be effective for local control of PanNENs (33), but in this case, multiple peritoneal disseminated nodules were noted without liver metastases. In cases with unresectable PanNEC, current guidelines advise selecting chemotherapeutic combination regimens, such as etoposide plus cisplatin or irinotecan plus cisplatin (34). Accordingly, chemotherapy was planned for this case, given the clinical diagnosis of unresectable PanNEC; however, it could not be introduced because of the rapid deterioration of the patient's general condition. If tolerable, early introduction of chemotherapy would have led to the patient's improved prognosis.

In conclusion, we have reported a case of PanNEC mimicking IPMC. Although rare, it should be noted that PanNEC, which correlates with the MPD and is accompanied by marked dilation of the MPD by highly viscous fluid production, does exist as one phenotype. Therefore, histological diagnosis is mandatory for the diagnosis of PanNEC with atypical imaging findings, and this case highlights that POPS-guided biopsy can be a useful diagnostic modality. However, the accumulation of additional data from more cases is necessary to further elucidate this type of PanNEC.

## Data Availability Statement

The original contributions presented in the study are included in the article/supplementary material, further inquiries can be directed to the corresponding author.

## Ethics Statement

The studies involving human participants were reviewed and approved by the Ethics Committee of the Kindai University Hospital. The patients/participants provided their written informed consent to participate in this study.

## Author Contributions

HT and KM wrote this manuscript, performed the endoscopic procedures, and cared for the patient. TH and KM analyzed and interpreted the imaging findings. MKi performed pathological examinations. YO, KK, KY, and MT reviewed the literature. TW and MKu critically revised the manuscript for intellectual content. All authors have read and approved the final manuscript.

## Conflict of interest

The authors declare that the research was conducted in the absence of any commercial or financial relationships that could be construed as a potential conflict of interest.

## Publisher's Note

All claims expressed in this article are solely those of the authors and do not necessarily represent those of their affiliated organizations, or those of the publisher, the editors and the reviewers. Any product that may be evaluated in this article, or claim that may be made by its manufacturer, is not guaranteed or endorsed by the publisher.
